# Arabic translation and validation of three knee scores, Lysholm Knee Score (LKS), Oxford Knee Score (OKS), and International Knee Documentation Committee Subjective Knee Form (IKDC)

**DOI:** 10.1051/sicotj/2018054

**Published:** 2019-03-08

**Authors:** Khamis Mohamed Ahmed, Hatem G. Said, Eslam Karam Allah Ramadan, Mohamed Abd El-Radi, Maher A. El-Assal

**Affiliations:** 1 Faculty of Medicine, Assiut University Assiut Egypt; 2 Department of Orthopedics and Truamatology, Assiut University Hospitals Assiut Egypt

**Keywords:** Lysholm Knee Score, Oxford Knee Score, International Knee Documentation Committee Subjective Knee Form, Knee injury and Osteoarthritis Outcome Score, translation, validation.

## Abstract

*Aim of the work*: Translation and validation of three commonly used knee scores to Arabic language: the Lysholm Knee Score (LKS), the Oxford Knee Score (OKS), and IKDC Subjective Knee Form.

*Methods*: Our work focused on translation and validation of the LKS, OKS and IKDC. Construct validity was assessed by comparing the LKS, OKS, and IKDC Subjective Knee Form and previous Arabic translated version of Knee injury and Osteoarthritis Outcome Score (KOOS). Test−retest reliability, internal consistency, and construct validity were assessed, using Intraclass Correlation Coefficient (ICC), Cronbach's alpha, and Pearson correlation coefficient (*r*).

*Results*: Reliability was excellent for the Arabic IKDC subjective form (0.95), while the Arabic LKS and the Arabic OKS were good: 0.8 and 0.85, respectively. The Cronbach's ά was excellent for the Arabic LKS and Arabic OKS: 0.9 and 0.90, respectively, while the Arabic IKDC subjective form was good (0.89). Construct validity was high for the Arabic LKS and the Arabic OKS: 0.7 and 0.913, respectively, while the Arabic IKDC was moderate (0.4) in cases of ACL and meniscus injuries and mild (0.18) in cases of osteoarthritis.

*Conclusion*: Arabic LKS and Arabic OKS were reliable and valid scores for patients complaining of ligamentous injuries, meniscus injuries, and osteoarthritis to be used for Arabic-speaking people, while the Arabic IKDC had excellent reliability and mild validity in cases of osteoarthritis and moderate validity in cases of ACL and meniscus injuries.

## Introduction

Questionnaires are important tools in orthopedic surgery in order to evaluate the impact of any surgical procedure on patients' daily life [1,2].

The major problem dealing with knee scores is their development in English language, so translation and validation of these scores into other language were mandatory [3,4].

Until now no valid translation of the LKS, OKS, and IKDC scores into Arabic language has been developed.

KOOS has already been developed into Arabic language in 2012 [5,6].

Cross-cultural adaptation protocols are necessary to adjust the health-related evaluation with languages to achieve excellent equality with the original form [7–10]. This operation indicates not only to the translation but also to the adaptation across the cultures, and adoption manners of different life [1,3,4].

The aim of our work was to translate and validate three of commonly used knee scores to Arabic version: LKS, OKS, and IKDC.

## Material and methods

### Translation

1. Translation of the original knee outcome score (English) into Arabic by two English translators.
2. Review of translations and synthesis of the first project (version 0.1).
3. Return to translation from Arabic to English for version 0.1 by two English translators.
4. Review of both the backward and forward translations. Formulation of the second version in Arabic (version 0.2) by a specialized language translator specializing in medical questionnaires and by a third translator.
5. Pretesting of the work (version 0. 2) by a group of 4 orthopedic surgeons and 30 patients to confirm that the draft could be understood [1,3,11].
6. Writing of version 1.0. after a few culture-related differences necessitated the use of some modifications to the original questions in order to suite the Arabic life style.

Patients in this study completed version 1.0 of these knee scores and statistical analysis of data was done upon this version 1.0. Patients were informed that their questions from these scores would be used for this study and informed consents were obtained. The patients were given Arabic version copy of the three knee outcome scores.

To establish reliability and construct validity, the scores were applied 15 days preoperative, 1 day preoperative, and 6 month post-operative and then compared with the KOOS that was previously translated and validated into Arabic language [1,5].

#### Patients

From March 2016 to November 2017, 100 patients with knee problems were recorded from the Assiut University Hospital, Egypt after pilot group. Our candidate inclusion criteria were ligamentous injuries, meniscus injuries, and osteoarthritis based on clinical and radiological findings by their orthopedic surgeon(s), age range was between 18 and 70 and the mean age was 38.7.

The patients were from Egypt and Arabic-speaking language with good education in order to understand and answer the questionnaire. Our candidate exclusion criteria were the refusal of patients to participate in the study and patients unable to read these scores.

#### Instruments

The Lysholm Knee Scale (LKS) is divided into eight sections that assess instability (25 marks), pain (25 marks), catching (15 marks), stair climbing (10 marks), swelling (10 marks), need for support (5 marks), squatting (5 marks), and limping (5 marks).

Each response question has been assigned a random scale on an increasing scale. The total score is the sum of each response to the eight questions and may range from 0 to 100. Higher results of the score indicate a better result with fewer symptoms ([12–14], Appendix).

The Oxford-12 knee score (OKS), published in 1998 [15–17], originally examined 12 items with a possible score of 1–5 for each. Scores thus ranged from 12 to 60, with 12 as the best result (Appendix).

The IKDC Subjective Knee Form was divided into three sections: (1) symptoms including swelling, pain, stiffness, giving way, and locking, (2) sports [3], (3) current knee function and knee function after knee injury (not included in the total score) [18]. Number of items of IKDC, 18 (7 items for symptoms, 1 item for sport activity, 9 items for daily activities, and 1 item for current knee function) (Appendix).

The KOOS consists of 42 items with five sections:

Symptoms (S), pain (P), sport, activities of daily living (ADL), and recreation (Sport/Rec), and quality of life related to the knee (QoL). The Likert scale was used from five points from 0 (no problem) to 4 (severe problems) to record each section and the scores from each unit were individually changed to 0 = 100 scale (0 = extreme knee problems, 100 = no knee problem) [5,10,19].

## Analysis of data

### Feasibility

It refers to the proportion of the patient who did not respond to any question according to the previous visit to surgery. The feasibility study was analyzed in 100 questionnaires completed on the first visit [1,20]. It was represented using the Bland−Altman plot.

### Reliability

The reliability of the retest was applied to the current study of the 100 patients who answered the initial translated version of three knee scores after 15 days of initial visit. The reliability was assessed by Intra class Correlation Coefficient (ICC). It was considered acceptable, if it was equal to or greater than 0.7 [1,5].

### Internal consistency

It refers to a function of number of subscales and covariation. Random error due to item selection modeled in this estimate of reliability of the instruments based on internal consistency is Cronbach's *ά* [1,21,22]. It is calculated using a two-way fixed effects model, which measures the agreement between items.

Cronbach's *ά* is usually considered acceptable if the value is 0.70 or above [1,5]. Internal consistency was analyzed in the 100 questionnaires completed in the first visit. If the value of Cronbach's *ά* was 0.7, it is considered fair, if it was 0.8, it is considered good, and if it was 0.9, it is considered excellent [1,21,23].

### Validity

It is a tool that measures the property being investigated. This was measured by comparing the results obtained in 100 completion surveys in 15 days preoperative, 1 day preoperative, and 6 months postoperative in both scales (three knee scores and KOOS) [1–3,12,13,20,22].

Construct validity was assessed through Pearson correlation coefficient (*r*) and it addressed the ability of whether the questionnaire measured what it was intended to measure [18] using the Spearman's rho [1,5]. Pearson correlations: *r* < 0.30 = low; 0.30 < *r* < 0.60 = moderate; *r* > 0.60 = high [1,5,22].

## Results

### Gender distribution

Of the 100 hundred included in the study, 55 cases (55%) were males while 45 cases were females (45%).

### Surgical procedure

**Figure 1:**
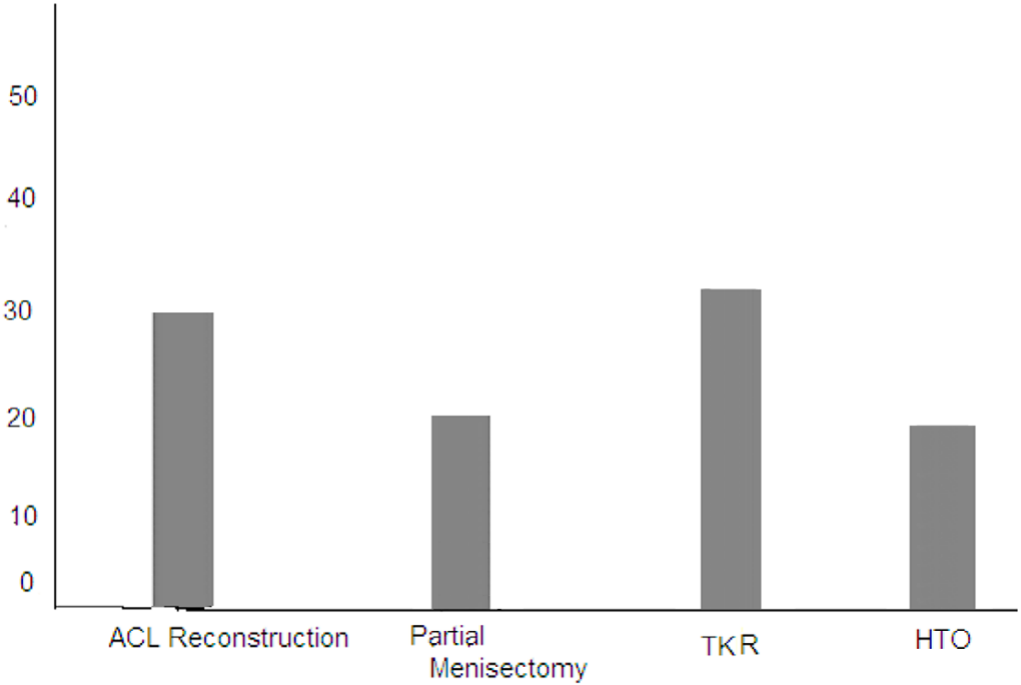
Distribution of surgical procedure.

Fifty cases underwent knee arthroscopy: 30 cases for ACL reconstruction and 20 cases for arthroscopic partial menisectomy, while the remaining 50 cases complain of OA and underwent TKR (30 cases) and HTO (20 cases) (Figure 1).

### Age distribution

The patient's age ranges between 18 and 70, and the mean age = 38.7 years.

**Table 1:** Differential age for various types of disease.

Type of disease	Range of age	Mean age
ACL tear	18–25	21.5
Meniscus tear	25–30	27.3
Osteoarthritis	40–70	50.7

### Feasibility

**A-LKS:** One hundred patients were studied for feasibility, of which 98 (98%) filled out the entire questionnaire, while 2 (2%) left question number 5 (locking) without answering.

**A-OKS:** One hundred patients were studied for feasibility, of which 95 (95%) filled out the entire questionnaire, while 5 (5%) left either question number 4 (How long can you walk before the pain from the knee becomes severe?) (with or without crutches) or question number 7 (Could you kneel down and get up afterward?) without answering.

**A-IKDC subjective form:** One hundred patients were studied for feasibility, of which 97 (97%) filled out the entire questionnaire, while 3 (3%) left either item number 6 (In past 4 weeks, or since injury, did knee catch?) or item number 7 (What is the level of activity that you can do without giving a clear knee way?) without answering.

### Reliability

ICC was excellent for the A-IKDC subjective form (0.95), while the A-LKS and A-OKS were good − (0.8) and (0.85), respectively.

### Internal consistency

Cronbach's *ά* was excellent for the A-LKS (0.9) and the A-OKS (0.90), while it was good for the A-IKDC subjective form (0.89).

### Construct validity

**A-LKS**: Showed high construct validity against the KOOS (0.7).

**A-OKS:** Showed high construct validity against the KOOS (0.913).

**A-IKDC:** Showed moderate construct validity against the KOOS (0.58). The samples of the patients were divided into two groups:

1. ACL and meniscus injuries: The construct validity was moderate (0.4).
2. Osteoarthritis: The construct validity was mild (0.18).

## Discussion

Orthopedic scoring evaluation is an important tool in the evaluation of treatment effectiveness in orthopedic surgery. Ideally any score should be reliable, valid, and practical.

Although the LKS, OKS, and IKDC scores are adopted and validated in many languages, there is no Arabic adoption and validation for these scores. In this study, we translated and adopted these scoring systems into the Arabic language for patients undergoing knee surgery (ACL reconstruction, menisectomy, HTO, and TKR).

In this study, a few culture-related differences necessitated the use of some modifications to the original questions in order to suit the Arabic life style. In the LKS, question 8 was modified by adding the inquiry about squatting during praying and eating on the ground, which is quite common among Arab population as well as farming. In the IKDC score, question 9 is modified by adding the inquiry about squatting in a manner similar to question 8 in the LKS. In the IKDC score, the low-demand sport in question 8 is defined as walking and bicycling rather than golf and bowling.

In other studies, during cross-cultural adaptation of the LKS into Chinese language [1,4], most patients found difficulty to understand the terms in the questionnaire, for example, “catching” and “instability”; therefore, the meaning of these terms was attached in simple language beside it the final version of the Chinese LKS during the pre-evaluation period. This was similar to cross-cultural adaptation of the IKDC subjective form into Korean language [4,24]. Authors have held a committee of experts several times on the cultural equivalence of cultural and linguistic aspects during intercultural adaptation as “giving way,” and “squatting” are common terms in English language. In contrast to the Korean language, these words were not found. Thus, the authors discussed some expressions that are composed of several words and can be easily understood among Koreans without changing the original meaning. In addition, Koreans are familiar with the metric system, so miles were converted to meters [24]. In contrast to the Portuguese LKS [25], questionnaire was easy to understand, especially that it was applied on individuals with good educational level, so there were no difficulties in reading it. Also, during the cross-cultural adaptation of the OKS into Finnish language [26], all participants deemed the questionnaire to be straightforward and easy to complete.

The results of the A-LKS and A-OKS were very good, no difficult questions, a few confusing items, and very low percentage of lost data for the items. These facts confirm that there are no translation problems, which is a reliable and valid measure for patients in Arabic-speaking countries with a variety of knee problems [5]. This is in contrast to the A-IKDC which had mild to moderate validity.

*Reliability* was good for the A-LKS (0.8) and A-OKS (0.85), while it was excellent for the A-IKDC subjective form (0.95). This is similar to the Greek IKDC (0.095) in patients with knee-related injuries [27], the Portuguese LKS (0.9) in patients complaining of ACL injuries [25], the Swedish OKS (0.94) in patients complaining of osteoarthritis [28], and the Chinese LKS (0.935) in patients complaining of ACL injuries [4]. It was good for the Finnish OKS (0.81) in patients complaining of osteoarthritis [26].This shows that the Arabic translation of LKS, OKS, and IKDC is reliable and this means that there is no difference between the test–retest values.

*The internal consistency* was accepted for all of the three scores. In this study, Cronbach᾿s *ά* for the A-LKS and A-OKS was excellent: 0.9 and 0.90, respectively. The internal consistency for the A-IKDC subjective form was good (0.89). This is similar to the Portuguese LKS (Cronbach's *ά* = 0.9) [25], the Turkish OKS (Cronbach's *α*: 0.90) in patients complaining of osteoarthritis [29], the Swedish OKS (Cronbach's *ά* = 0.93) [28], and the Greek IKDC (Cronbach's *α* = 0.87) [27]. In contrast, the Korean IKDC (K-IKDC) was excellent (Cronbach's *α* = 0.91) [24]. This indicates that there is a strong relationship regarding the data collected in the first visit.

*The construct validity* of the A-LKS showed high construct validity against the KOOS (0.7) similar to the construct validity of the Chinese LKS in patients complaining of ACL injuries against the IKDC and WOMAC (*r* = 0.837) [4]; the A-OKS also showed high construct validity against the KOOS (0.913) similar to the correlation between the Finnish OKS and the RAND-36 questionnaire and KOOS (*r* = 0.913) [26]. In contrast to the construct validity for the Turkish OKS against the WOMAC, SF-36 scores showed a significant correlation (*r* < 0.05) [29].

The A-IKDC subjective form showed mild construct validity against the KOOS (*r* = 0.18) in cases of osteoarthritis. It showed a moderate construct validity against the KOOS (*r* = 0.4) in the cases of ACL and meniscus injuries. The explanation for this result might be that the IKDC is mainly planned for sports injuries rather than osteoarthritis. This is confirmed by the low pre-operative and post-operative scores, as the IKDC is most useful to evaluate patients presented with ACL injury [30]. The construct validity of A-IKDC in cases of ACL and meniscus injuries is only moderate. The explanation for this result might be that the IKDC has many questions and with some repetitions that confuse the patients. This is similar to the correlation between the Greek IKDC and the SF-36 (*r* = 0.60) in patients with knee-related injuries [27].

*The limitation* in our study was the lack of comparison to other Arabic versions of knee questionnaires besides the KOOS that would have allowed us to better assess the construct validity.

## Conclusion

The A-LKS and A-OKS are reliable and valid scores for patients suffering from ligamentous injuries, meniscus injuries, and osteoarthritis. While the A-IKDC has excellent reliability and mild validity in cases of osteoarthritis and moderate validity in cases of ACL and meniscus injuries. These scores are a good outcome tool for use in Arabic-speaking countries.

## Conflict of interest

The authors declare that they have no conflicts of interest in relation to this article.
