# Correction Notice to: Arabic translation and validation of three knee scores, Lysholm Knee Score (LKS), Oxford Knee Score (OKS), and International Knee Documentation Committee Subjective Knee Form (IKDC)

**DOI:** 10.1051/sicotj/2019023

**Published:** 2019-08-01

**Authors:** Khamis Mohammed Ahmed Ali, Hatem G. Said, Eslam Karam Allah Ramadan, Mohamed Abd El-Radi, Maher A. El-Assal

**Affiliations:** 1 Faculty of Medicine, Assiut University Assiut Egypt; 2 Department of Orthopedics and Truamatology, Assiut University Hospitals Assiut Egypt

An error occurred in the article, the name of one author “Mohamed Abd El-Radi” was missing.

The new version of the article contains the corrected and complete list of authors.

The publisher apologizes for this mistake.

